# Factors associated with cervical precancerous lesions among women screened for cervical cancer in Addis Ababa, Ethiopia: A case control study

**DOI:** 10.1371/journal.pone.0191506

**Published:** 2018-01-19

**Authors:** Hirut Teame, Adamu Addissie, Wondimu Ayele, Selamawit Hirpa, Alem Gebremariam, Gdiom Gebreheat, Ahmedin Jemal

**Affiliations:** 1 Department of Public Health, College of Medicine and Health Sciences, Adigrat University, Adigrat, Ethiopia; 2 School of Public Health, College of Health Sciences, Addis Ababa University, Addis Ababa, Ethiopia; 3 Department of Nursing, College of Medicine and Health Sciences, Adigrat University, Adigrat, Ethiopia; 4 Surveillance & Health Services Research, American Cancer Society, Atlanta, Georgia, United States of America; University of Kentucky, UNITED STATES

## Abstract

**Background:**

Cervical cancer is the second most prevalent cancer among women in the developing countries including Ethiopia. Precancerous lesions can be developed and risk to the development of cervical cancer over time. Early identification of the precancerous lesion and its risk factor is paramount in preventing cervical cancer. However, the determinants of cervical precancerous lesions are not well documented in Ethiopia. Therefore, this study is conducted to determine factors associated with cervical precancerous lesion among women screened for cervical cancer.

**Methods:**

A hospital-based unmatched case-control study was conducted in selected health facilities in Addis Ababa from March to April 2016. Data were collected from 114 cases and 229 controls using an interviewer-administered questionnaire, entered to Epi Info version 7, and exported to SPSS version 20 for analysis. Odds ratios with its 95% confidence intervals and two-tailed P-value were calculated. Variables with P-value ≤ 0.2 in the bivariate analysis were included in the multivariate logistic regression model.

**Results:**

Women aged 40–49 years had 2.4-fold higher odds of precancerous lesions compared to those aged 30–39 (Adjusted Odds Ratio = 2.4, 95% Confidence Interval: 1.27–4.54). Women having history of sexually transmitted infections were significantly associated with cervical precancerous lesion compared to their counterparts (Adjusted Odds Ratio = 3.20, 95% Confidence Interval: 1.26–8.10). Similarly, those women who had two or more lifetime sexual partners (Adjusted Odds Ratio = 2.17 95% Confidence Interval: 1.01–4.67), and women whose husbands had two or more lifetime sexual partners (Adjusted Odds Ratio = 3.03, 95% Confidence Interval: 1.25, 7.33) had higher odds of cervical precancerous lesions.

**Conclusions:**

Older age, history of multiple sexual partners and sexual transmitted infections were associated with increased risk of precancerous lesion. Therefore, women with higher risk of precancerous lesions should be encouraged to be screened more frequently for cervical cancer.

## Introduction

Worldwide, there were 528,000 cases and 266,000 deaths of cervical cancer in 2012. The incidence is higher among developing countries. According to Globocan, there were about 7,095 newly diagnosed cervical cancer cases and 4,732 cervical cancer deaths in 2012 in Ethiopia, accounting for about 17.0% of total cancer cases and deaths in females. It is the second most commonly diagnosed cancer as well as the second leading cause of cancer-related deaths in women in Ethiopia [1].

Cervical cancer is usually developed after prolonged phase of pre-invasive lesions in the cervix [2]. Therefore, early identification and treatment at its pre-invasive stage may benefit the clients and decrease the burden of morbidity and mortality resulting from cervical cancer [3, 4].

Visual Inspection with Acetic Acid (VIA) is one of the screening modality of cervical precancerous lesion [5]. Screening with VIA in resource limited settings is commonly preferable method than Human Papilloma Virus (HPV) test and cytologic or Pap smear. This is because it does not need more advanced testing requirements (trained cytotechnicians or pathologists and other programmatic requirement) [6]. Recently, in Ethiopia cervical cancer screening centers are being established to provide screening services for all eligible women [7]. However, screening uptake in the community is low [8, 9] because of lack of awareness of the community on cervical cancer risk factors and prevention methods [9–13]. Further, screening uptake as well as knowledge about cervical cancer risk factors and prevention are also low among the health workers [14, 15].

Identifying factors associated with the cervical precancerous lesion is important for planning a more targeted screening programs to decrease the high morbidity and mortality of the disease in the country. However, the risk factors for cervical precancerous lesions among the general population in the setting of Ethiopia are not well identified. Studies conducted so far in Ethiopia are limited to assessing the cost and its predictors of cervical cancer treatment, prevalence and predictors of pap smear cervical epithelial cell abnormality, risk factors associated with invasive cervical carcinoma, knowledge about cervical cancer, HPV prevalence, prevalence and risk factors among Human Immune Deficiency Virus (HIV) positive women [11, 16–19]. The Ethiopian health sector development program IV (2010/11–2014/15) includes the prevention and control of cancer [20]. Based on this policy guidance and the global and national epidemiologic situation, the federal Ministry of Health of Ethiopia has recently designed and developed a national guideline on prevention and control for cervical cancer [7]. We undertook this research to identify factors associated with cervical precancerous lesion in order to inform and strengthen the existing cervical cancer prevention and control programs in the country. In addition, the study will inform health education about cervical cancer prevention and stimulate etiologic research about cervical precancerous lesions.

## Methods

This hospital-based unmatched case-control study was conducted in Addis Ababa, the capital city of Ethiopia. According to the central statistics authority estimate for 2015, the population of Addis Ababa was 3,384,569, of whom 52.4% were females [21]. The city has 11 governmental hospitals, 31 private hospitals and nongovernmental clinics, and 89 health centers.

Sexually active women aged 21–49 years who had undergone screening for cervical precancerous lesion using VIA were included in the study. Cases were women with positive visual inspection with acetic acid findings of acetowhite lesions around the squamocolumnar junction of the cervix. Controls were women with negative visual inspection with acetic acid findings of acetowhite lesion around the squamocolumnar junction [6]. Sample size was calculated using Epi info version 7 considering the parameters of 95% level of confidence, 80% power, taking a 1:2 ratio of cases to controls, and a 41.3% proportion of multiple sexual partners among general population [21]. After adding 10% non-response rate, the final sample size become 120 cases and 240 controls.

### Sampling procedures

Out of the 131 total health facilities in Addis Ababa, 11 provide cervical cancer screening service. The study was conducted in 6 of those health facilities. These health facilities were selected purposely, with the selection criteria being provision of cervical cancer screening using visual inspection with acetic acid regularly for all women regardless of HIV status and greater than one year of establishment. The selected health facilities were; Kolfe sub city Woreda 09 health center, Family Guidance clinic, Zewditu hospital, Kolfe health center, Lideta health center and Mary Joy clinic. The total sample size was allocated to each selected health facility using probability proportionate to the average monthly client flow, as reviewed from registration book.

The study subjects were identified from those screened with visual inspection with acetic acid. All women with positive visual inspection with acetic acid screens within three months before and during the data collection period were included in the study until the required sample size was obtained. Those cases screened within three months before data collection time were identified from the registration book, and were summoned by telephone for face-to-face interview at the health facilities where they were screened. Controls were selected alternatively from women negative for visual inspection with acetic acid who screened during data collection period.

### Data collection procedures

An interviewer-administered semi-structured questionnaire was adapted by reviewing similar studies [17, 19, 22]. The questionnaire includes questions about the socio-demographic and economic characteristics, reproductive, lifestyle and sexual behavior of the study participants (See S1 File). The questionnaire was first prepared in English, then translated to Amharic and pre-tested. Data were collected by 6 trained nurses who were providing cervical cancer screening service at the time of data collection. All questionnaires were checked for completeness every day by the principal investigator and supervisor.

Routine cervical cancer screening was provided to all women by trained nurses. According to the World Health Organization (WHO) guidelines for screening and treatment of precancerous lesions for cervical cancer prevention, the result can be interpreted as: positive, when an acetowhitish lesion with well-defined margins observed with in the vicinity of the transformation zone, or if the whole cervix turned white (visual inspection with acetic acid—positive); negative when there is no acetowhitish lesion (visual inspection with acetic acid—negative); or suspicious for cancer when there is visible ulcerative cauliflower-like ulcer, oozing and bleeding on touch [6]. Woman with findings of “suspicious” were not included in the study (N = 13).

### Data analysis

Data were entered to Epi Info version 7 then transported to Statistical Package for Social Science (SPSS) version 20. Data were cleaned by running simple frequency distributions, summary statistics and cross tabulation. Descriptive statistical methods were used to summarize socio-demographic and clinical characteristics of the study participants. Odds ratios with 95% confidence intervals and two-tailed P-values were calculated to identify the presence and strength of association. Variables with P-value ≤ 0.2 in the bivariate analysis were included into a multivariate logistic regression analysis.

Participants’ age, educational status, occupation, monthly income, parity, history of abortion, previous family history of cervical cancer, history of previous screening for cervical cancer, condom use, history of pelvic infection, ever had history of Sexual Transmitted Diseases (STD), ever had history of STD in their sexual partner, participants’ number of lifetime sexual partners, and number of lifetime sexual partners of the participants’ husband were included in the multivariate model to assess the individual effect of each variable on the outcome of interest. In the logistic regression, the final model was fitted using enter method. Statistical significance was declared at P-value < 0.05.

### Ethical statement

Ethical clearance was obtained from Addis Ababa University, College of Health Sciences, School of Public Health Research Ethics Committee with the registration number of SPH/020/08. An ethical clearance form was written to each health institution from Addis Ababa Regional Health Bureau. The respondents were informed about the objective and purpose of the study, and verbal consent was obtained from the study subjects. During the pretest, participants preferred to give verbal consent than written consent. This is a common research practice in the country [23, 24]. After the participants provided their verbal consent, the data collector signs the consent form on behalf of the participant ensuring the participants’ willingness to participating in the study. (See S1 File). Moreover, this consent process was approved by the Addis Ababa University School of Public Health Research Ethics Committee. Participants who were summoned to the facility for an interview were compensated for transportation cost.

## Results

A total of 120 cases and 240 controls were enrolled, of whom 17 women (6 cases and 11 controls) refused to participate, for a non-response rate of 4.7%. The reported reason for non-participation was lack of time for interview. The prevalence of cervical precancerous lesions among women screened for cervical cancer was 12.8%.

Among the study participants, 70 (61.40%) of the cases and 84 (36.68%) of the controls were found to be in the age group of 40–49 years old. The mean and standard deviation of the age of cases and controls were 42.26 ± 6.50, and 37.48 ± 7.55 years, respectively. Thirty-two (28.07%) of the cases and 94 (41.05%) of the controls had a bachelor’s degree or above (Table 1).

**Table 1:** Socio-demographic characteristics of women screened for cervical cancer in Addis Ababa, Ethiopia, 2016.

Variables	Cases, n (%)	Controls, n (%)	COR (95% CI)	P-value
**Age (years)**				
21–29	11 (9.65)	44 (19.21)	0.76(0.36–1.65)	0.50
30–39	33 (28.95)	101 (44.10)	1.00	
40–49	70 (61.40)	84 (36.68)	2.55(1.54–4.23)	0.00
**Educational status**				
No formal education	28 (24.56)	40 (17.47)	1.00	
Primary education	33 (28.95)	60 (26.20)	0.79 (0.41–1.50)	0.46
Secondary/preparatory	21 (18.42)	35 (15.28)	0.86 (0.42–1.77)	0.68
College or above	32 (28.07)	94 (41.05)	0.49 (0.26–0.91)	0.02
**Marital status**				
Single	4 (3.51)	5 (2.18)	1.00	
Married	90 (78.95)	187 (81.66)	0.60 (0.16–2.29)	0.46
Widowed	6 (5.26)	14 (6.11)	0.54 (0.10–2.72)	0.45
Divorced	14 (12.28)	23 (10.04)	0.76 (0.17–3.32)	0.72
**Occupation**				
Housewife	61 (53.51)	119 (51.96)	1.00	
Merchant	12 (10.53)	9 (3.93)	2.60 (1.04–6.51)	0.04
Daily laborer	10 (8.77)	17 (7.42)	1.15 (0.50–2.66)	0.75
Governmental employee	16 (14.04)	45 (19.65)	0.69 (0.36–1.33)	0.27
Private/NGO employee	15 (13.16)	39 (17.03)	0.75 (0.38–1.47)	0.40
*Income per month (USD)				
≤ 44.04	34 (29.82)	46 (20.09)	1.00	
44.08–132.11	45 (39.47)	100 (43.67)	0.61 (0.35–1.07)	0.09
132.16–220.19	26 (22.81)	57 (24.89)	0.62 (0.32–1.17)	0.14
≥220.19	9 (7.90)	26 (11.35)	0.47 (0.20–1.13)	0.09
**Religion**				
Orthodox Christian	72 (63.16)	161 (70.30)	1.00	
Muslim	24 (21.05)	34 (14.85)	1.58 (0.87–2.85)	0.13
Protestant or Jehovah Witness	18 (15.80)	34 (14.85)	1.18 (0.63–2.24)	0.60

*USD: US dollar

At the time of this study, 90 (78.95%) of the cases and 187 (81.66%) of the controls were married, and 4 (3.51%) of the cases and 5 (2.18%) of the controls were single. By occupation, more than half 61 (53.51%) of the cases and 119 (51.96%) of the controls were housewives, followed by governmental employee 16 (14.04%) and 45 (19.65%) of the cases and controls respectively. The income per month ranged from 17.61 to 880.74 USD, with a median of 110.09 USD. Thirty-four (29.82%) of the cases and 46 (20.09%) of the controls had an average monthly income less than 44.04 USD. Seventy-two (63.16%) of the cases and 161 (70.30%) of the controls were Orthodox Christian (Table 1).

### Reproductive health characteristics

The rate of current contraceptive use was 26.9% and 22.7% among the cases and controls, respectively. The magnitude of ever contraceptive use was 62.28% and 62.88% among the cases and controls, respectively. The dominant contraceptive method used was injectable, followed by pills. Among the study subjects, 68 (59.65%) of the cases and 146 (63.76%) of the controls had regular menstrual history. The mean age at menarche was similar between cases and controls, 13.93 and 14.24 years, respectively. Twelve (10.53%) of the cases and eleven (4.80%) of the controls had history of post coital bleeding. Age at first birth for most respondents was less than 20 years. There was no significant difference in age at first birth between the cases and controls. Thirty-four (41.46%) of the cases and 52 (33.77%) of the controls had average birth interval less than two years. Among the participants, 11 (9.65%) of the cases and 12 (5.24%) of the controls had history of abortion greater than three times; 10 (8.77%) of the cases and 11 (4.80%) of the controls had a family history of cervical cancer (Table 2).

**Table 2:** Reproductive health related characteristics of screened women in Addis Ababa, Ethiopia, 2016.

Variables	Case n (%)	Control n (%)	COR (CI 95%)	P-value
**Pill use**				
No	85 (74.56)	181 (79.04)	1.00	
<5 years	21 (18.42)	30 (13.10)	1.49 (0.81–2.76)	0.20
≥5 years	8 (7.02)	18 (7.86)	0.95 (0.40–2.26)	0.90
**Injectable use**				
No	68 (59.65)	148 (64.63)	1.00	
<5 years	30 (26.32)	60 (26.20)	1.09 (0.64–1.84)	0.75
≥ 5 years	16 (14.04)	21 (9.17)	1.66 (0.81–3.38)	0.16
**Implant use**				
No	94 (82.46)	195 (85.15)	1.00	
<5 years	14 (12.28)	25 (10.92)	1.07 (0.52–2.18)	0.86
≥ 5 years	6 (5.26)	9 (3.93)	1.37 (0.47–3.96)	0.56
**IUCD use**				
No	102 (89.47)	198 (86.46)	1.00	
<5 years	9 (7.90)	15 (6.55)	1.16 (0.49–2.75)	0.73
≥ 5 years	3 (2.63)	16 (6.99)	0.36 (0.10–1.28)	0.12
**Age of menarche**				
≤ 12 years	15 (13.16)	21 (9.17)	1.00	
13–14 years	59 (51.75)	108 (47.16)	0.76 (0.37–1.59)	0.47
≥ 15 years	40 (35.09)	100 (43.67)	0.56 (0.26–1.19)	0.13
**Menstrual history**				
Regular	68 (59.65)	146 (63.76)	1.00	
Sometimes irregular	20 (17.54)	24 (10.48)	1.79 (0.92–3.46)	0.08
Always irregular	17 (14.91)	32 (13.97)	1.14 (0.59–2.20)	0.69
No menses	9 (7.90)	27 (11.79)	0.72 (0.32–1.60)	0.42
**Post coital bleeding**				
Yes	12 (10.53)	11 (4.80)	2.33 (1.00–5.46)	0.05
No	102 (89.47)	218 (95.20)	1.00	
**Parity**				
No	13 (11.40)	50 (21.83)	0.53 (0.26–1.06)	0.07
1–3	54 (47.37)	110 (48.04)	1.00	
≥ 4	47 (41.23)	69 (30.13)	1.39 (0.85–2.27)	0.19
**Age at first birth**				
<20 years	49(48.52)	89 (49.72)	1.00	
20–25 years	33 (32.67)	52 (29.05)	1.15 (0.66–2.02)	0.62
25–30 years	15 (14.85)	30 (16.76)	0.91 (0.45–1.85)	0.79
>30 years	4 (3.96)	8 (4.47)	0.91 (0.26–3.17)	0.88
**Average birth interval**				
<2 years	34 (41.46)	52 (33.77)	1.00	
2–3 years	28 (34.15)	53 (34.42)	0.81 (0.43–1.52)	0.51
>3 years	20 (24.39)	49 (31.82)	0.62 (0.32–1.23)	0.17
**History of abortion**				
No	47 (41.23)	126 (55.02)	1.00	
1–3	56 (49.12)	91 (39.74)	1.65 (1.03–2.65)	0.04
≥ 4	11 (9.65)	12 (5.24)	2.46 (1.02–5.95)	0.05
**Family history of cervical cancer**				
Yes	10 (8.77)	11 (4.80)	1.91 (0.78–4.63)	0.16
No	104 (91.23)	218 (95.20)	1.00	

### Lifestyle and sexual behavior

Prior to the index screening, 28(24.35%) of the cases and 40(17.47%) of the controls had ever been screened for cervical cancer. Only 10 (35.71%) of the cases and 10 (25.00%) of the controls had a positive history of cervical precancerous lesion. Seven (6.14%) of the cases and nine (3.93%) of the controls have history of smoking. The majority of the cases (79.82%) and controls (78.60%) had never used condom in their lifetime. The mean and standard deviation of age at first marriage was 19.57 ± 4.13 for cases and 20.35 ± 5.10 for controls. Six of ten (61.52%) of the respondents started their first sex at the age of 18 and above years old. Thirty-four (29.82%) of the cases and 39 (17.03%) of the controls had a history of pelvic infection. Besides, 39 (34.21%) of the cases and 23 (10.04%) of the controls had history of sexual transmitted diseases (STD). The magnitude of history of STD was higher in cases (19.30%) compared to controls (6.11%). Similarly, the magnitude of HIV was higher in the cases (40.71%) than the controls (23.45%). The magnitude of having lifetime multiple sexual partner among cases and controls was 71 (62.28%) and 70(30.57%), respectively. Similarly, about one fourth of the participants’ husbands had had two or more other lifetime sexual partners (Table 3).

**Table 3:** Lifestyle and sexual behavior characteristics of screened women in Addis Ababa, Ethiopia, 2016.

Variables	Case n (%)	Control n (%)	OR (95%CI)	P-value
**Previously screened for cervical cancer**				
Yes	28 (24.35)	40 (17.47)	1.47 (0.85–2.54)	0.17
No	87 (75.65)	189 (82.53)	1.00	
**Time since last screening**				
<1 year	18 (64.29)	19 (47.50)	1.00	
1–3 years	7 (25.00)	15 (37.50)	0.49 (0.16–1.49)	0.21
>3 years	3 (10.71)	6 (15.00)	0.53 (0.11–2.43)	0.41
**Result of the last screen (for cervical pre-cancerous lesions)**				
Positive	10 (35.71)	10 (25.00)	1.67 (0.58–4.78)	0.34
Negative	18 (64.28)	30 (75.00)	1.00	
**Ever history of smoke**				
Yes	7 (6.14)	9 (3.93)	1.60 (0.58–4.41)	0.36
No	107 (93.86)	220 (96.07)	1.00	
**Age at first marriage**				
< 15 years	19 (17.27)	38 (16.96)	1.00	
15–17 years	17 (15.45)	32 (14.28)	1.06 (0.48–2.38)	0.88
≥18 years	74 (67.27)	154 (68.75)	0.96 (0.52–1.78)	0.90
**Age at first sex**				
<15 years	26 (22.81)	44 (19.21)	1.00	
15–17 years	22 (19.30)	40 (17.47)	0.93 (0.46–1.90)	0.84
≥ 18 years	66 (57.90)	145 (63.32)	0.77 (0.44–1.36)	0.37
**Condom use**				
Always	9 (7.90)	9 (3.93)	1.00	
Sometimes	14 (12.28)	40 (17.467)	0.35 (0.12–1.06)	0.06
Never	91 (79.82)	180 (78.60)	0.51 (0.19–1.32)	0.16
**Ever history of pelvic infection**				
Yes	34 (29.82)	39 (17.03)	2.07 (1.22–3.51)	0.01
No	80 (70.18)	190 (82.97)	1.00	
**Ever history of STD**				
Yes	39 (34.21)	23 (10.04)	4.66 (2.61–8.31)	0.00
No	75 (65.79)	206 (89.96)	1.00	
**Ever history of STD in sexual partner**				
Yes	22 (19.30)	14 (6.11)	3.67 (1.80–7.49)	0.00
No	92 (80.70)	215 (93.89)	1.00	
**Ever HIV tested**				
Yes	113 (99.12)	226 (98.69)	1.50 (0.15–14.58)	0.73
No	1 (0.88)	3 (1.31)	1.00	
**HIV status**				
Positive	46 (40.71)	53 (23.45)	2.24 (1.38–3.64)	0.00
Negative	67 (59.29)	173 (76.55)	1.00	
**Lifetime sexual partners**				
One	43 (37.72)	159 (69.43)	1.00	
Two or above	71 (62.28)	70 (30.57)	3.75 (2.34–6.01)	0.00
**Other lifetime sexual partners of the husband**				
No	32 (28.07)	124 (54.15)	1.00	
One	33 (28.95)	66 (28.82)	1.94 (1.10–3.43)	0.02
Two or above	49 (42.98)	39 (17.03)	4.87 (2.75–8.63)	0.00

### Factors associated with cervical precancerous lesions

Those variables tested with p-value ≤ 0.2 in the bivariate logistic regression analysis were entered into multivariate logistic regression analysis (Table 4). Controlling for the effect of other confounding factors, age group, history of STD, lifetime sexual partners of the women, and having other life-time sexual partners of the husband were found to be significantly associated with precancerous cervical cancer.

**Table 4:** Multivariate analysis of selected variables among study participants of Addis Ababa, Ethiopia, 2016.

Variables	Case n (%)	Control n (%)	COR (CI 95%)	AOR (CI 95%)
**Age (years)**				
21–29	11 (9.65)	44 (19.21)	0.76 (0.36–1.65)	1.00 (0.40–2.50)
30–39	33 (28.95)	101 (44.10)	1.00	1.00
40–49	70 (61.40)	84 (36.68)	2.55 (1.54–4.23)	2.40 (1.27–4.54)*
**Educational status**				
No formal education	28 (24.56)	40 (17.47)	1.00	1.00
Primary education	33 (28.95)	60 (26.20)	0.79 (0.41–1.50)	0.61 (0.27–1.40)
Secondary/preparatory	21 (18.42)	35 (15.28)	0.86 (0.42–1.77)	0.86 (0.31–2.35)
College or above	32 (28.07)	94 (41.05)	0.49 (0.26–0.91)	0.56 (0.21–1.50)
**Occupation**				
House wife	61 (53.51)	119 (51.96)	1.00	1.00
Merchant	12 (10.53)	9 (3.93)	2.60 (1.04–6.51)	2.36 (0.76–7.33)
Daily laborer	10 (8.77)	17 (7.42)	1.15 (0.50–2.66)	0.83 (0.26–2.63)
Governmental employee	16 (14.04)	45 (19.65)	0.69 (0.36–1.33)	1.10 (0.46–2.65)
Private/NGO employee	15 (13.16)	39 (17.03)	0.75 (0.38–1.47)	0.63 (0.26–1.53)
**Income per month**				
≤ 1000	34 (29.82)	46 (20.09)	1.00	1.00
1001–3000	45 (39.47)	100 (43.67)	0.61 (0.35–1.07)	0.52 (0.25–1.10)
3001–5000	26 (22.81)	57 (24.89)	0.62 (0.32–1.17)	0.47 (0.20–1.14)
≥5000	9 (7.90)	26 (11.35)	0.47 (0.20–1.13)	0.70 (0.21–2.28)
**Parity**				
No	13 (11.40)	50 (21.83)	0.53 (0.26–1.06)	0.51 (0.21–1.26)
1–3	54 (47.37)	110 (48.04)	1.00	1.00
>3	47 (41.23)	69 (30.13)	1.39 (0.85–2.27)	1.46 (0.77–2.78)
**History of abortion**				
No	47 (41.23)	126 (55.02)	1.00	1.00
1–3	56 (49.12)	91 (39.74)	1.65 (1.03–2.65)	0.80 (0.44–1.48)
>3	11 (9.65)	12 (5.24)	2.46 (1.02–5.95)	1.59 (0.56–4.51)
**Family history of cervical cancer**				
Yes	10 (8.77)	11 (4.80)	1.91 (0.78–4.63)	2.38 (0.82–6.92)
No	104 (91.23)	218 (95.20)	1.00	1.00
**Previous screened for cervical cancer**				
Yes	27 (23.68)	40 (17.47)	1.47 (0.85–2.54)	1.02 (0.51–2.06)
No	87 (76.32)	189 (82.53)	1.00	1.00
**Condom use**				
Always	9 (7.90)	9 (3.93)	1.00	1.00
Sometimes	14 (12.28)	40 (17.467)	0.35 (0.12–1.06)	0.34 (0.08–1.40)
Never	91 (79.82)	180 (78.60)	0.51 (0.19–1.32)	0.41 (0.12–1.46)
**Ever history of pelvic infection**				
Yes	34 (29.82)	39 (17.03)	2.07 (1.22–3.51)	1.75 (0.92–3.32)
No	80 (70.18)	190 (82.97)	1.00	1.00
**Ever history of STD**				
Yes	39 (34.21)	23 (10.04)	4.66 (2.61–8.31)	3.20 (1.26–8.10)*
No	75 (65.79)	206 (89.96)	1.00	1.00
**Ever history of STD in sexual partner**				
Yes	22 (19.30)	14 (6.11)	3.67 (1.80–7.49)	1.34 (0.41–4.38)
No	92 (80.70)	215 (93.89)	1.00	1.00
**HIV status**				
Positive	46 (40.71)	53 (23.45)	2.24 (1.38–3.64)	1.26 (0.64–2.50)
Negative	67 (59.29)	173 (76.55)	1.00	1.00
**Lifetime sexual partners**				
One	43 (37.72)	159 (69.43)	1.00	1.00
Two or above	71 (62.28)	70 (30.57)	3.75 (2.34–6.01)	2.17 (1.01–4.67)*
**Other lifetime sexual partners of the husband**				
No	32 (28.07)	124 (54.15)	1.00	1.00
One	33 (28.95)	66 (28.82)	1.94 (1.10–3.43)	1.16 (0.54–2.49)
Two or above	49 (42.98)	39 (17.03)	4.87 (2.75–8.63)	3.03 (1.25–7.33)*

* Significantly associated with cervical precancerous lesion

Women in the age group of 40–49 years were two times more likely to have cervical precancerous lesion than those who were 30–39 years (adjusted odds ratio = 2.40, 95% CI (1.27–4.54)) (Table 4).

Women who had a history of STD were three times more likely to have cervical precancerous lesion than those who did not (adjusted odds ratio = 3.20, 95% CI (1.26–8.10)). Women who had two or more lifetime sexual partners were significantly associated with cervical precancerous lesions (adjusted odds ratio = 2.17, 95% CI (1.01–4.67)). Having a husband with two or more other lifetime sexual partners was also significantly associated with cervical precancerous lesions (adjusted odds ratio = 3.03, 95% CI (1.25–7.33)) (Table 4).

## Discussion

This study revealed that 12.8% of the women were positive for precancerous cervical lesion. Study subjects who were aged 40–49 years had higher odds of developing cervical precancerous lesions compared to those aged 30–39 years. This is similar with the study conducted in Addis Ababa, which reported that the peak incidence of cervical cancer was in the 40–49 years age group [25]. It is also similar to the findings of a study conducted in Jimma, Ethiopia, that found older age (40–59 years) to be at greater risk for invasive cervical cancer compared to those less than 40 years [11]. The age difference among the women screened for cervical cancer could be due to the longer period for potential exposure to the HPV virus and due to the time required for a cervical precancerous lesion to develop [5]. However, there are also studies which documented findings contradicting with this finding [19, 26]. Jean D et al., documented age as a protective factor [26] whereas, Gessesse et al. documented the absence of statistically significance between precancerous lesion and age of the women [19]. These discrepancies could be explained in part by differences in the characteristics of study population given that the studies by Gessesse et al and Jean et al [19, 26] were conducted among HIV positive women whereas our study was conducted among all women in the reproductive age group. The discrepancies may also reflect differences in sample size of the studies.

We found that odds of history of STD was three times as high in women with cervical precancerous lesion than in the control group. Multiple studies in different settings have revealed that having history of sexual transmitted diseases is a risk factor for developing cervical precancerous lesions [16, 17, 27–30]. This association could be the result of HPV, which is the common cause of both STD and cervical precancerous lesion [31].

Having two or more lifetime sexual partners was also found to be significantly associated with cervical precancerous lesion, presumably because an increase in number of sexual partners raises the risk of HPV infection [32]. This finding is supported by multiple studies conducted in Ethiopia [11, 16, 17, 19].

Similarly, our finding of the significant association between the subject’s husband or partner with two or more other lifetime sexual partners and increased risk of cervical precancerous lesion was consistent with previous findings [11]. Specifically, a study conducted in Southwest Ethiopia reported that having husband who had more than one wife in his lifetime was a risk factor for invasive cervical cancer. These associations are plausible given these women have a higher risk of acquiring HPV infection, which is the causative agent for cervical precancerous lesion and cervical cancer [33, 34].

In this study cases and controls were identified only via their current VIA result; associated with the validity of this screening test, there could be misclassification of cases and controls. This might contribute to underestimating the estimation of cause effect relationship between some of the explanatory variables and the outcome of interest, cervical precancerous lesion. Moreover, though we have restricted the interview to the newly screened women, recall bias could affect their response. We have used trained data collectors however; the role of social desirability bias in the response of the participants could be anticipated.

## Conclusions

This study confirms that older age (40–49 years), history of sexual transmitted diseases, and history of having multiple sexual partners are risk factor for precancerous cervical lesions. Therefore, it is important to consider these risk factors in designing an early screening program for cervical cancer. Cervical cancer screening program in Ethiopia may target women above the age of 40 years, and those having risky sexual behavior in view of limited resources and infrastructure for a large-scale screening program.

## Supporting information

S1 File Questionnaire. (DOCX)

## Abbreviations

ACS: American Cancer Society
AOR: Adjusted Odds Ratio
ART: Anti-Retro-Viral Therapy
COR: Crude Odds Ratio
HIV: Human Immunodeficiency Virus
HPV: Human Papilloma Virus
OR: Odds Ratio
STD: Sexual Transmitted Diseases
SPSS: Statistical Package for Social Science
VIA: Visual Inspection with Acetic Acid
WHO: World Health Organization
IUCD: Intra Uterine Contraceptive Device
