# Supplementary material for: Factors associated with cervical precancerous lesions among women screened for cervical cancer in Addis Ababa, Ethiopia: A case control study
Source: PLoS One. 2018 Jan 19;13(1):e0191506. doi: 10.1371/journal.pone.0191506 (PMC5774809; doi:10.1371/journal.pone.0191506)
Supplement: S1 File — (DOCX) [file pone.0191506.s001.docx]

| **English version questionnaire**I: Information sheet Addis Ababa University College of Health Sciences School of Public Health information sheet on the factors associated with cervical precancerous lesion among women screened for cervical cancer in Addis Ababa city.  Hello! How are you? My name is __________.I live in this city. Now I am a research team member to be conducted here by a post graduate student in public health in Addis Ababa university, supervised by School of Public Health instructors. The purpose of the study is assessing the factors associated with cervical precancerous lesion among women screened for cervical cancer in this city. If you agree to participate in the study as respondent, you will not have any risk in participating in the study except the time you spent during the interview. The study may be advantageous in identifying risk factors for cervical precancerous lesion, so it is important to develop strategies that help to improve the prevention and control methods of cervical cancer. All the genuine information obtained from you will be strictly kept confidential, your participation is surely voluntary, and no monetary incentives will be given for your participation in the study. You can withdraw any time during conducting the study, also your participation, non-participation, or refusal to answer questions will not have any effect on your life, and your name will not be recorded on this form. If you have any question Mrs Hirut Teame is the contact person. Hirut can be reached through a call at 0946415072.  Are you willing to participate in the interview and stay with us for few minutes (15-20) now?  [ ] Yes, Go to next page [ ] No, Thanks! Proceed to next eligible participant  **Note:** Women who undergo screening for cervical precancerous lesion, who are sexually active, women, who are from 21 to 49 years of age and women who live in Addis Ababa city for the last six months. II: Consent form |  |  |  |  |  |  |
| --- | --- | --- | --- | --- | --- | --- |

I am informed that my identity and the information I give will be treated confidentially. I have also been informed that I can refuse to participate in the study or not to respond to questions if I am not interested. Furthermore, I have been informed that I can stop responding to the questions at any time in the process. I am informed that my participation, non-participation, or refusal to answer questions will not have any effect on my life. I am informed that no monetary incentives will be given for my participation in the study. I am also informed that my response will be used to develop strategies that help to improve the prevention and control methods of cervical cancer.

If the study subject agrees to participate in the study, thank her and start the interview.

Interviewer signature certifying that informed consent has been given verbally by the respondent.

Interviewer‘s name---------------------------- Signature-------------------Date---------------------

**Note:** No need of enforcing the clients to be included in the study.

Thank you!

## III: Questionnaire (English Version)

**General information**

For each question, make a circle around the spelling that corresponds to the answer; fill the blanks with the answer or mark “x”.

1. Participant’s code number: ____________

**CASE CONTROL**

**Part 1: Socio-demographic characteristics**

| S.No | Question | Response | Skip |
| --- | --- | --- | --- |
| 1.1 | How old are you? (completed years) | ___________ |  |
| 1.2 | What is your level of education? | 1. Don’t write and read 2. Only read and write 3. Primary (1-4) 4. Primary (5-8) 5. Secondary (9-10) 6. Preparatory 7. Diploma or technical/vocational 8. Higher (bachelor degree and above) |  |
| 1.3 | What is your marital status? | 1. Single 2. Married   C. Widowed  D. Divorced  E. Separated |  |
| 1.4 | How old were you when you first marriage? (If she is already married once) | ______________ |  |
| 1.5 | How much is your family average monthly income(ETB) | _______________ |  |
| 1.6 | What is your current occupation status? | A. House wife  B. Merchant  C. Daily laborer  D. Governmental employee  E. Private/NGO employee  F. Others (specify__________) |  |
| 1.7 | What is your religion? | A. Orthodox Christian B. Muslim  C. Protestant D. Catholic  E. Others(specify__________) |  |

**Part 2: Questions related to reproductive health factors**

| 2.1 | Do you ever use contraceptive? | 1. Yes B. No | 2.4 |
| --- | --- | --- | --- |
| 2.2 | If answer for Q 2.4 yes, which type of contraceptive do you use? (you can choose more than one choice) | A. Pill  B. Injectable (Depo)  C. Implant  D. Others (specify _____________________) |  |
| 2.3 | For how long have you been using contraception? |  |  |
| 2.4 | How old were you when you menarche? | ___________ |  |
| 2.5 | How was your menstrual history? | A. Regular  B. Sometimes irregular  C. Always irregular  D. No menses, why ____________ |  |
| 2.6 | Have you ever-experienced post coital bleeding? | 1. Yes B. No |  |
| 2.7 | Have you ever give birth? | 1. Yes B. No | 2.11 |
| 2.8 | If answer for Q 2.12 yes, how many times? | ___________ |  |
| 2.9 | How old were you when you first birth? | _________ |  |
| 2.10 | What is the average birth interval between your births? (if she has two or more births) | _________ |  |
| 2.11 | Have you ever-experienced abortion? | 1. Yes B. No | 2.13 |
| 2.12 | If answer for Q 2.17 yes, how many times? | ___________ |  |
| 2.13 | Do you have family (mother or sister) history of cervical cancer? | 1. Yes B. No |  |
| **Part 3: Questions related to lifestyle and sexual behavior factors** | | |  |
| 3.1 | Have you ever been screened for cervical cancer before? | 1. Yes B. No | 3.4 |
| 3.2 | If answer for Q 3.1 yes, when were you screened for the last time? | _____________ |  |
| 3.3 | What was the result of that screening test? | A. Positive B. Negative |  |
| 3.4 | Have you ever smoke? | A. Yes B. No | 3.6 |
| 3.5 | If yes, how long you have been smoke? | ____________ |  |
| 3.6 | How old were you when you first had sex? | ____________ |  |
| 3.7 | Do you use condom whenever you are having sex? | 1. Always B. Sometimes   C. Never |  |
| 3.8 | Have you ever been told you that you had a pelvic infection or treated by health professionals? | A. Yes B. No |  |
| 3.9 | Have you had a sexually transmitted infection in your lifetime? | A. Yes B. No |  |
| 3.10 | Does your partner ever have history of STIs? | A. Yes B. No |  |
| 3.11 | Do you ever have history of genital ulcer or swelling? | A. Yes B. No |  |
| 3.12 | Does your partner ever have history of genital ulcer or swelling? | A. Yes B. No |  |
| 3.13 | Have you been tested for HIV before? | 1. Yes B. No | 3.16 |
| 3.14 | If answer for Q 3.20 yes, what was the result? | 1. Positive B. Negative   C. Unknown |  |
| 3.15 | If answer for Q 3.21 positive, did you start antiretroviral therapy? | 1. Yes B. No |  |
| 3.16 | How many sexual partners have you had in your lifetime? | ___________ |  |
| 3.17 | Does your partner have other partners? | A. Yes B. No |  |
| 3.18 | If answer for Q 3.24 yes, how many? | ___________ |  |

**Thank you!**

**ክፍል አንድ: መረጃ መስጫ ወረቀት**

ኣዲስ ኣበባ ዩኒቨርስቲ ጤና ሳይንስ ኮሌጅ የጤና አጠባበቅ ትምህርት ቤት የማህፀን ጫፍ ቕድመ ካንሰር ጠንቆች በሴቶች በኣዲስ ኣበባ ከተማ የሚደረግ ጥናት ነው።

ጤና ይስጥልኝ፤ ስሜ -------------------------እይባላል፡፡ እኔ በአዲስ አበባ ዩኒቨርሲቲ የጤና አጠባበቅ ትምህርት ቤት የማስተርስ ድግሪ የምታጠና ተማሪ ከአዲስ አበባ ዩኒቨርሲቲ መምህራንጋ በመታገዝ በአዲስ አበባ የማህፀን በር ቅድመ ካንሰር ተያያዥ ምክንያቶች ለማጥናት በተዋቀረው ቡድን ውስጥ አባል ነኝ፡፡ ጥናታችንም ወደ ጤና ተቋማት ለቅድመ ካንሰር ምርምራ በመጡ ሴቶች በመጠየቅ የሚከናወን ነው፡፡ እርሶም በጥናት ቡድን አማካኝነት ጥናቱ ላይ ተሳታፊ እንዲሆኑ ተመርጠዋል፡፡ እርሶ የሚሰጡትን መረጃ ከሌሎች ምንጮች ጋር ተዳምሮ የማህፀን በር ቅድመ ካንሰር ተያያዥ ምክንያቶች ለይቶ ለማወቅ ወይም የሚሻሻልበት ሁኔታ ለመፍጠር ታልሞ የተዘጋጀ ጥናት ነው፡፡ በሂደታችን ውስጥ በጥናቱ ላለመካፈል በማኛውም ወቅት ከወሰኑ በማኛውም ሰዓት መጠይቁን እናቆማለን፡፡ በጥናቱ ውስጥ ላለመካፈል በሚወስኑት ውሳኔ የተነሳ የሚደርስቦት አንዳችም ሁኔታ የለም፡፡ በቃለ መጠይቁ ወቅት የሚሰጡት መረጃዎች ለጥናቱ ዓላማ ብቻ የሚውሉና ሚስጢራዊነቱ ሙሉ በሙሉ የተጠበቀ ነው፡፡ በዚህ መጠይቅ ውስጥ ስሞትንና እርሶን ለመለየት የሚያገለግል ነገር አይጻፍም፡፡ ቃለ መጠየቁ የሚወስድብዎት ግዜ ከ 15-20 ደቂቃ ብቻ ነው፡፡ ግልጽ ያልሆነ ነገር ካለ ሊጠይቁን ይችላሉ፡፡ ማንኛውም ጥያቄ ካሎት የጥናቱ መሪ የሆኑትን ወ/ሮ ሂሩት ጠዓመ በስልክ ቁጥር 251946415072 ማግኘት ይችላሉ፡፡ ስለተባበሩን እናመሰግናለን፡፡

በቃለ መጠይቁ ተስማምቻለሁ_________ ወደ የስምምነት ቅጽ ይለፉ

በቃለ መጠይቁ አልተስማማሁም _______ አመስግነው በዚህ ያብቁ

**አስታውስ**፤ ተሳታፊዋ የማህፀን በር ቅድመ ካንሰር ምርምራ ያደረገች፣ ግብረ ስጋ ግንኙነት ማድረግ የጀመረች፣ ከ 21-49 ዕድሜ ክልል ውስጥ፣ ቀዋሚ የመኖሪያ ቦታ አዲስ አበባ መሆን ኣለባቸው ፡፡

**ክፍል ሁለት: የስምምነት ቅጽ**

ተመራማሪዋ የጥናቱን አላማ በሚገባ ግልጽ በሆነ ቋንቋ አስረድተውኛል፡፡ በዚህም መሰረት የጥናቱን አላማ ስለተረዳሁ ለመሳተፍ ውሳኔዬን በሚከተለው መንገድ አረጋግጣለሁ፡፡

የመረጃ ሰብሳቢ ፊርማ በቃል ስምምነት መስጠቱን ያረጋግጣል፡፡

የመረጃ ሰብሳቢ ስም --------------------------- ፊርማ --------------- ቀን ------------

**አስታውስ**፤ ተሳታፊዋ በግድ በጥናቱ እንዲሳተፍ አያስገድዱ፡፡

**ክፍል ሶስት: ቃለ መጠይቅ**

የተሳታፊዋ መለያ ቁጥር _______

የማህፀን በር ቅድመ ካንሰር ያላት [ ] የማህፀን በር ቅድመ ካንሰር የሌላት [ ]

**ክፍል I. የማህበራዊ: ኢኮኖሚያዊና ዲሞግራፊያዊ ሁኔታዎች**

| ቁጥር | ጥያቄ | ምላሽ | ዝለል |
| --- | --- | --- | --- |
| 1.1 | ዕድሜ | ________ |  |
| 1.2 | የትምህርት ደረጃዎ? | ሀ. መፃፍና ማንበብ የማትችል  ለ. መፃፍና ማንበብ ብቻ የምትችል  ሐ. የመጀመሪያ ደረጃ (1-4)  መ. የመጀመረያ ደረጃ(5-8)  ረ. ሁለተኛ ደረጃ (9-10)  ሰ. መሰናዶ# ቴክኒክ እና ሙያ (10^+^)  ሸ. ዲፕሎማ  ቀ. ከፍተኛ (ዲግሪ እና ከዛ በላይ) |  |
| 1.3 | የጋብቻ ሁኔታዎ? | ሀ. ያላገባች  ለ. ያገባች  ሐ. ባሏ የሞተባት  መ. የተፋታች  ረ. ተለያይተው የሚኖሩ |  |
| 1.4 | የመጀመሪያ ጋብቻ ሲፈጽሙ እድሜዎት ስንት ነበር?(ያገቡ ከሆነ) | ________________ |  |
| 1.5 | ጠቅላላ የቤተሰብዎ ወርሀዊ ገቢ ስንት ነው? | የብሩን መጠን _____________ |  |
| 1.6 | ስራዎ ምንድን ነው? | ሀ. የቤት እመቤት  ለ. ነጋዴ  ሐ. ቀን ሰራተኛ  መ. የመንግስት ሰራተኛ  ረ. መንግስታዊ ያልሆነ  ሰ. ሌላ__________ |  |
| 1.7 | ሐይማኖት | ሀ. ኦርቶዶክስ  ለ. ሙስሊም  ሐ. ፕሮቴስታንት  መ. ካቶሊክ  ረ. ሌላ__________ |  |

**ክፍል II: ስለ ተዋልዶ ጤና ጥያቄዎች**

| 2.1 | የወሊድ መከላከያ ተጠቀመው ያውቃሉ? | ሀ. አዎ  ለ. አልጠቀምም | 2.4 |
| --- | --- | --- | --- |
| 2.2 | የወሊድ መከላከያ እየተጠቀሙ ከሆነ ወይም ከነበረ የትኛውን ዓይነት ነው የሚጠቀሙት? (ከአንድ በላይ መምረጥ ይቻላል) | ሀ. የሚዋጥ ፒል  ለ. በመርፌ የሚሰጥ  ሐ. በክንድ የሚቀበረውን  መ. በማህጸን ውስጥ የሚቀመጥ  ረ. ሌላ__________ |  |
| 2.3 | ለምን ያህል ግዜ ተጠቀሙ? (ከአንድ በላይ እየተጠቀሙ ከነበሩ ለሁሉም ይፃፉ) | _______________________________ |  |
| 2.4 | በስንት ዓመትዎ ነው የመጀመሪያውን የወር አበባ ያዩት? | ___________ |  |
| 2.5 | የወር አበባዎ ዑደት እንዴት ነው? | ሀ. በየወሩ በትክክል ይመጣል  ለ. አንዳንዴ ይዛባል  ሐ. ብዙ ግዜ ይዛባል  መ. የወር አበባ አላይም ካሉ ለምን________________ |  |
| 2.6 | ከግብረ ስጋ ግንኙነት በኋላ ደም የማየት ነገር አሎት? | ሀ. አዎ ለ. የለኝም |  |
| 2.7 | ልጅ ወልደዋል? | ሀ. አዎ ለ. አልወለድኩም | 2.11 |
| 2.8 | አዎ ካሉ ምን ያህል ልጅ ወልደዋል? | _________ |  |
| 2.9 | በስንት አመትዎ ነው የመጀመሪያውን ልጅ የወለዱት? | _________ |  |
| 2.10 | በኣማካይ በልጆችዎ መካከል ያለ የእድሜ ልዩነት ስንት ነው?(ሁለት እና ከዛ በላይ ልጅ ከወለደች) | _________ |  |
| 2.11 | ውርጃ ኖሮት ያውቃል ? | ሀ. አዎ ለ. አያውቅም | 2.13 |
| 2.12 | አዎ ካሉ ስንት ግዜ? | ___________ |  |
| 2.13 | በቤተሰብ የማህፀን ካንሰር ያለበት ሰው አለ? | ሀ. አዎ ለ. የለም |  |
| **ክፍል III: ስለ ግል ባህርያት የሆኑት ጥያቄዎች** | | |  |
| 3.1 | ከዚህ በፊት የማህፀን ጫፍ ካንሰር ተመርምረው ያውቃሉ? | ሀ. አዎ ለ. አላውቅም | 3.4 |
| 3.2 | ለመጨረሻ ግዜ የተመረመሩት መቼ ነው? | _________________ |  |
| 3.3 | የምርመራው ውጤቱ ምን ነበር? | ሀ. ፖዘቲቭ ለ. ነጋቲቭ |  |
| 3.4 | ሲጃራ ኣጭሰው ያውቃሉ? | ሀ. አዎ ለ. አላጨስም | 3.6 |
| 3.5 | አዎ ካሉ ለምን ያህል ግዜ አጨሱ? | ___________ |  |
| 3.6 | ግብረ ስጋ ግንኙነት ለመጀመሪያ ግዜ ማድረግ ሲጀመሩ እድሜዎት ስንት ነበር? | ____________ |  |
| 3.7 | ግብረ ስጋ ግንኙነት በሚያደርጉበት ግዜ ኮንዶም ይጠቀማሉ? | ሀ. ሁሌ  ለ. አልፎአልፎ  ሐ. አልጠቀምም |  |
| 3.8 | የማህፀን ኢንፌክሽን አለቦዎት ተብለው ወይም ታክመው ያውቃሉ? | ሀ. አዎ  ለ. አላውቅም |  |
| 3.9 | የአባላዘር በሽታ አለብዎት ተብለው ወይም ታክመው ያውቃሉ? | ሀ. አዎ  ለ. አላውቅም |  |
| 3.10 | ባለቤትዎ ወይም የፍቅር ጓደኛዎ የአባለዘር በሽታ አለበት ተብለው ያውቃሉ? | ሀ. አዎ  ለ. አያውቅም |  |
| 3.11 | በራስዎ ብልት አካባቢ ላይ የሚያሳክክ ጠባሳ ወይም ዕብጠት ወጥቶቦት ያውቃል? | ሀ. አዎ  ለ. አያውቅም |  |
| 3.12 | ባለቤትዎ (የፍቅር ጓደኛዎ) ብልት አካባቢ ላይ የሚያሳክክ ጠባሳ ወይም ዕብጠት ወጥቶበት ያውቃል? | ሀ. አዎ  ለ. አያውቅም |  |
| 3.13 | የኤች አይ ቪ ምርመራ አድርገው ያውቃሉ? | ሀ. አዎ ለ. አላውቅም | 3.16 |
| 3.14 | ከተመረመሩ ውጤቱ ምን ነበር? | ሀ. ፖዘቲቭ  ለ. ነጋቲቭ  ሐ. አይታወቅም |  |
| 3.15 | ካለዎት የኤች አይ ቪ መድሃኒት ጀመሩ? | ሀ. አዎ  ለ. አልጀመርኩም |  |
| 3.16 | እስከ አሁን ድረስ ከስንት ወንዶች ጋር ግብረ ስጋ ግንኙነት አድርገው ያውቃሉ? | ___________ |  |
| 3.17 | ባለቤትዎ(የፍቅር ጓደኛዎ) ከሌላ ግብረ ስጋ ግንኙነት ኣለው? | ሀ. አለው  ለ. የለውም |  |
| 3.18 | አለው ካሉ ከምን ያህል ሰው? | ___________ |  |

ስለ ሰጡኝ ምላሽ በጣም አመሰግናለሁ!
